# Systemic Transcriptional Alterations of Innate and Adaptive Immune Signaling Pathways in Atherosclerosis, Ischemia Stroke, and Myocardial Infarction

**DOI:** 10.4172/1948-593X.1000120

**Published:** 2015-04-03

**Authors:** Taura L. Barr, Reynal L. VanGilder, Ryan Seiberg, Ashely Petrone, Paul D. Chantler, Chiang-Ching Huang

**Affiliations:** 1 Department of Health Restoration School of Nursing and Center for Basic and Translational Stroke Research West Virginia University, USA; 2 Department of Health Restoration West Virginia University School of Nursing and Emergency Medicine, USA; 3 Feinberg School of Medicine, Northwestern University, USA; 4 School of Medicine, Center for Neuroscience West Virginia University, USA; 5 Division of Exercise Physiology and Center for Cardiovascular and Respiratory Sciences, School of Medicine, West Virginia University, USA; 6 Joseph J. Zilber School of Public Health, University of Wisconsin, Milwaukee, USA

**Keywords:** Atherosclerosis, Stroke, Gene expression, Immune system

## Abstract

**Background:**

Transcriptional profiles are available for a variety of cardiovascular-related diseases. The goal of this study was to compare blood transcriptional profiles of the Toll-like receptor (TLR), T-cell receptor (TCR), and B-cell receptor (BCR) signaling pathways in asymptomatic atherosclerosis, acute ischemic stroke, and myocardial infarction patients to identify common mechanisms of immune regulation and their association with epigenetic regulation.

**Methods and results:**

Peripheral blood gene expression profiles from human atherosclerosis-related diseases and healthy controls were downloaded from Gene Expression Omnibus (GEO). Genes in the TLR, TCR, and BCR pathways were retrieved from the NCBI BioSystems database. Significance of gene enrichment and concordance of expression changes in each pathway was compared between studies. Gene expression was significantly correlated across the three disease conditions (p<10^−15^) and the proportion of significant genes was high (30~60%, p<0.001). Hub genes identified by weighted gene co-expression network analysis (WGCNA) in the TCR/BCR sub-network, including CD81 and TCR-CD3ζ, were significantly down-regulated and highly correlated with DNA (cytosine-5-)-methyltransferase 1 (DNMT1).

**Conclusion:**

Common biologically relevant networks associated with immune regulation in stroke, atherosclerosis, and myocardial infarction were discovered. Given the high correlation of DNMT1 with these immune signaling pathways, epigenetic regulation may contribute to the coordination of innate and adaptive immune response in all CVD disease states. Down-regulation of the TCR-BCR axis in the adaptive immune system offers critical information for the investigation of the functional mechanisms underlying chronic inflammation-induced immune suppression in cardiovascular disease and stroke.

## Introduction

Atherosclerosis is one of the most common causes of death and disability in the United States [1]. Atherosclerosis is a complex process associated with the accumulation of plaque and arterial remodeling. The atherosclerotic process involves an ongoing, interaction between chronic inflammation and altered immune function [2,3]. The intertwined determinants of the disease require the use of analytic strategies that can simultaneously assess the contribution of multiple genes to the onset and progression of atherosclerosis [4–6].

In most clinical cases, ischemic stroke (IS) and myocardial infarction (MI) result from long standing atherosclerotic disease [7], thus it can be expected that similarities in immune regulation exist among the three disease states. In fact, previous studies using gene expression profiling indicate that increased expression of genes involved in the toll-like receptor (TLR) signaling pathway are common in patients with asymptomatic atherosclerotic cardiovascular disease [4] and acute IS [5]. However, there are clinical differences in disease pathology between atherosclerosis, IS and MI. Experimental studies show that activation of TLRs can induce diverse inflammatory responses through specific epigenetic modification [8]. Remodeling of DNA methylation is a prominent epigenetic mechanism of adaptive immune responses, resulting in progressive loss of transcriptional adaption of T cells [9–12]. Therefore, it is likely that epigenetic modification may play a key role in coordinating the innate and adaptive immune response. Despite these novel discoveries, the interplay between DNA methylation and immune signaling pathways in atherosclerotic diseases has largely been unexplored outside the controlled laboratory setting. Furthermore, the molecular pathophysiology linking atherogenesis, immune dysregulation, epigenetic alteration, and risk for IS and MI is virtually unknown. This information may lead to improved diagnostic strategies to identify patients with atherosclerotic disease at high risk for IS and/or MI.

One strategy to address these gaps is to examine transcriptomic data of methylation regulation and immune signaling pathways through blood transcriptional profiling. The purpose of this study was to determine the extent of overlap in transcriptional patterns for TLR, TCR, and BCR signaling pathways and DNA methyltransferase 1 (DNMT1), a key molecule controlling DNA methylation pattern, among asymptomatic atherosclerotic, acute IS, and MI patients. We have found a significant overlap in expression among immune pathways in three cardiovascular disease states, with novel information suggesting that epigenetics may contribute to immune suppression in all three disease states. The detection of these significant transcriptional patterns may provide insights into the mechanisms of chronic inflammation-induced immune suppression in atherosclerosis-related diseases that can be further studied at the bench.

## Materials and Methods

Gene expression profiles of peripheral whole blood in human atherosclerosis-related diseases were downloaded from Gene Expression Omnibus (GEO): Atherosclerosis (GSE20129), Ischemic stroke (GSE16561), myocardial infarction (GSE29111). The characteristics of study samples in GSE 20129 and GSE16561 have been described previously [4,5]. Study GSE29111 includes two time points (7 and 28 hours) after MI for each of the 18 patients. The raw expression data were normalized by quantile normalization for the Illumina microarray chips (GSE20129 and GSE16561) and the Robust Multiarray, averaging method for the Affymetrix microarray chips (GSE29111). Genes in the toll-like receptor (TLR, 106 genes), T-cell receptor (TCR, 104 genes), and B-cell receptor (BCR, 72 genes) signaling pathways were retrieved from the NCBI BioSystems database [13] (BSID 83076, 83080, 83081). For genes with multiple probes or probesets, those with the most significant difference in expression between cases and controls or between 7 days and 28 days after MI events (for GSE 29111) were selected for study. A permutation test was used to evaluate the statistical significance of gene enrichment in each signaling pathway for each microarray study. Pearson correlation analysis was used to evaluate the similarity of expression changes across studies. Weighted gene co-expression network analysis (WGCNA) was used to identify gene modules and hub genes in the TLR/TCR/BCR pathways [14]. The false discovery rate (FDR) was calculated to account for multiple testing to identify pairs of genes with significant topological overlap [15]. To investigate common epigenetic control of immune response among the disease states, Pearson correlation analysis was used to examine the relationships between the expression of DNMT1 and all of the genes in the TLR, TCR, and BCR pathways across the studies.

## Results

**Figure 1:**
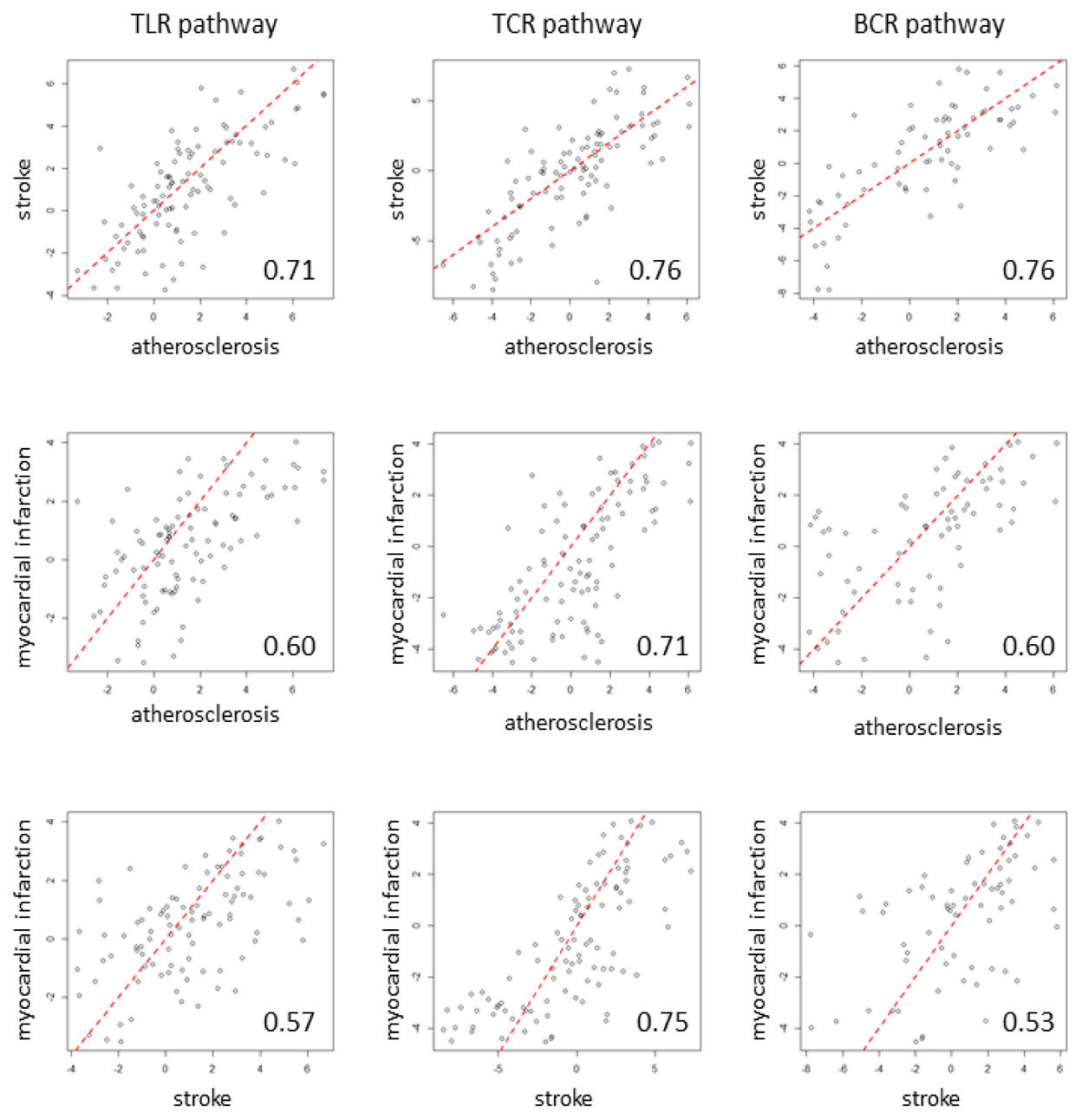
Concordance of expression changes (indicated by t statistics) in the TLR, TCR, and BCR pathways between cases and controls in atherosclerosis and stroke studies or between 7 hours and 28 hours after myocardial infarction. The number in each panel is the Pearson correlation coefficient. The red dash line is the identity line.

We sought to advance the understanding of common immune regulatory networks in patients with IS, MI, and subclinical atherosclerosis. There was high correlation among all three immune pathways and disease states. Differentially expressed genes were enriched in all three immune pathways in atherosclerosis, stroke, and myocardial infarction (p<0.001) (Table 1). Additionally, the changes of gene expression, indicated by t statistics, in all three pathways across the studies were highly concordant. The correlation of t statistics between each pair of studies ranged from 0.71–0.76 for TCR pathways, 0.57–0.71 for TLR pathway, and 0.53 to 0.76 for BCR pathway (p<10^−15^) (Figure 1).

**Figure 2:**
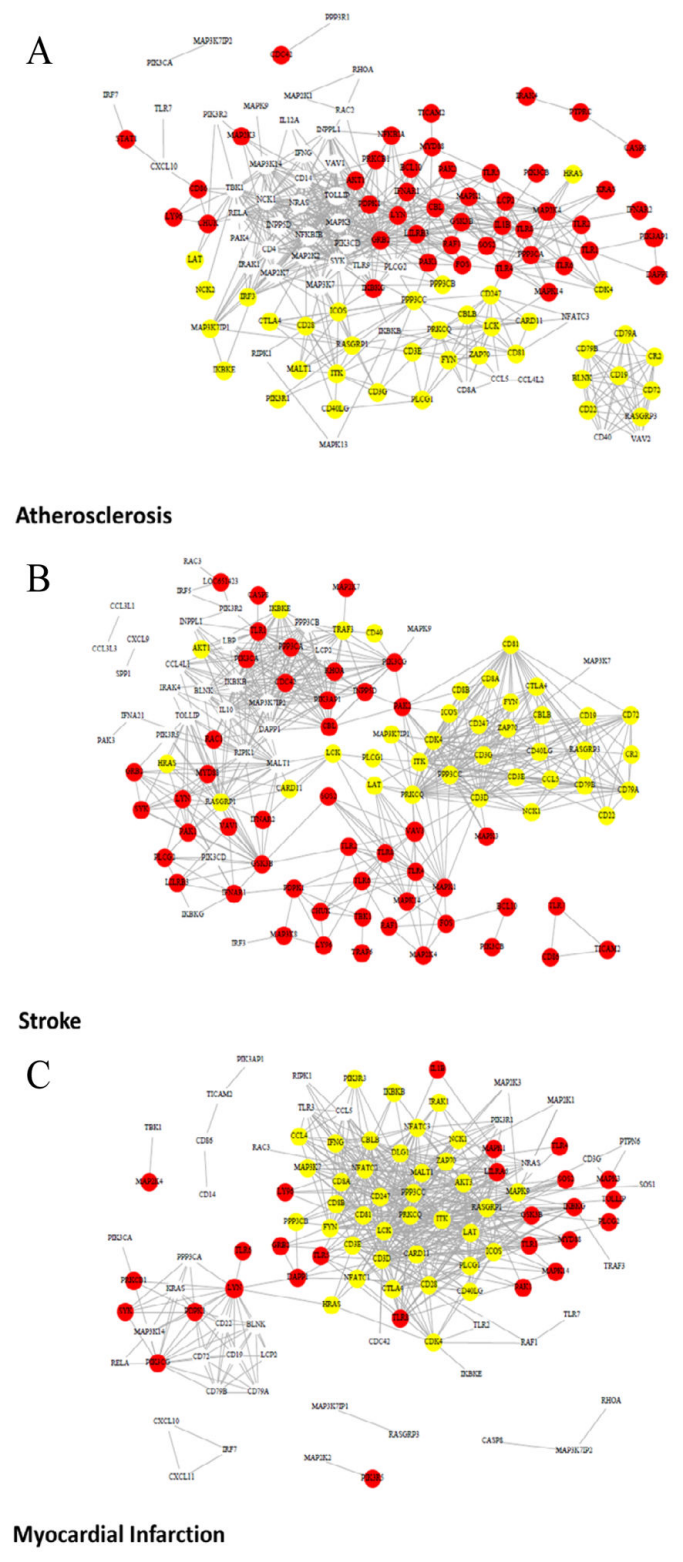
Topology-overlap matrix (TOM)-based connectivity network in the TLR, TCR, and BCR signaling pathways. Top 3% of gene pairs with largest TOM-based measures were selected to generate these networks. Red (yellow) dot: gene with significant increase (decrease) in expression in cases; circle: gene that is not differentially expressed between cases and controls. A: atherosclerosis; B: Ischemic Stroke; C: Myocardial Infarction.

**Figure 3:**
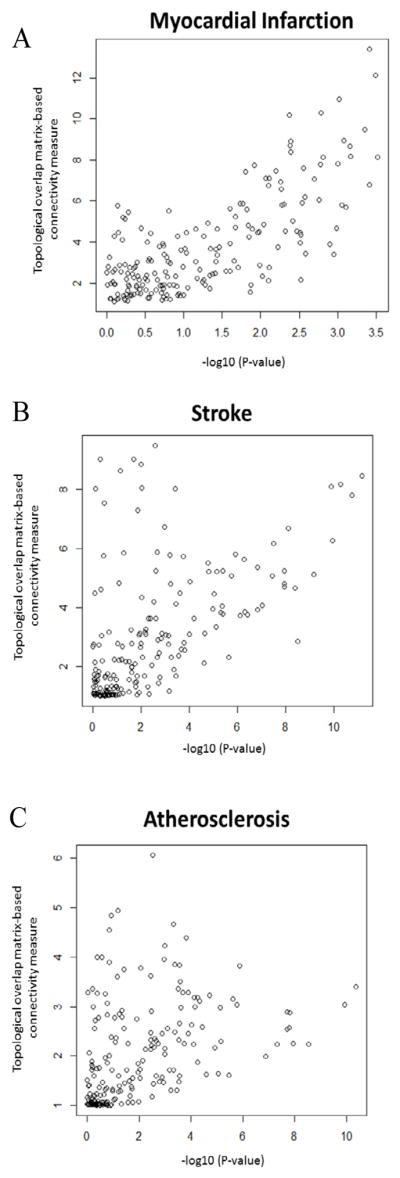
Correlation of significance of differential expression (x-axis) and TOM-based connectivity measures (y-axis) in atherosclerosis, ischemia stroke, and myocardial infarction studies. Pearson correlation is 0.74 for MI (A), 0.57 for IS (B), and 0.42 (C) for atherosclerosis.

We identified specific features of the immune network structure using the topological overlap matrix (TOM)-based connectivity measure. As expected, genes in the three immune pathways were highly connected in all three disease conditions. To illustrate the TOM-based connectivity networks, for each disease condition we selected the top 3% of pairs of genes with the highest topological overlap (FDR<0.1%) as shown in Figure 2. A consistent pattern of these networks among the three disease conditions was found in which two major sub-networks contained genes with decreased expression in the TCR pathway and genes with increased expression in the TLR pathway. The TCR subnetwork featured molecules in TCR-CD3ζ complex and CD81, while the TLR sub-network highlighted several TLR receptors (Figure 2). A third sub-network was highlighted by molecules that are crucial in BCR signaling, including CD79A, CD79B, CD19, CD22, and CD72. This sub-network also demonstrated significantly decreased expression in atherosclerosis and stroke, but not in myocardial infarction. As the TOM-based connectivity measure of a gene reflects the level of its overlap with other genes, we hypothesized that high TOM-based connectivity is associated with the significance of gene expression change between disease and control as indicated by −*log*_10_(*p*), where *p* denotes the p-value from a t test. This was indeed the case for the myocardial infarction data (Figure 3A: correlation 0.74, p<0.00001). We noticed that CD81 and genes in TCR-CD3 complex were among those with the highest TOM-based connectivity (Table S1). Such an association remained strong (correlation: 0.57, p<0.00001) for the stroke data; however, a bifurcation trend was observed (Figure 3B). The p-values, although still less than a 0.05 level, were relatively larger for some genes with the largest TOM-base connectivity measures. We found that most of these genes were downstream of the TCR or BCR signaling pathways (Table S1). For the atherosclerosis data, the positive association was less evident, albeit still significant (Figure 3C; correlation 0.42, p<0.0001).

In light of the inverse relationship of expression pattern among genes that control the innate and adaptive immune systems, we sought to test whether epigenetic regulation is associated with immune regulation. We found that genes that were most correlated with DNMT1 were molecules that control and sustain T- and B-cell activation, including CD81, CD247 (CD3 zeta), ITK, CD3D, LAT, CTLA4, CD79B (Table S1). In line with the finding above, DNMT1 is most correlated with the hub genes in the TCR and BCR sub-networks. In general, DNMT1 expression was negatively correlated with TLR signaling while positively correlated with TCR/BCR signaling. In addition, DNMT1 expression was significantly decreased in patients with IS, atherosclerosis and MI (P=6•10^−6^ for atherosclerosis; P=6•10^−8^ for IS; P=8•10^−5^ for MI).

## Discussion

In this study we compared peripheral blood transcription patterns of three major immune signaling pathways from patients with subclinical atherosclerosis, IS, and MI. We found a consistent pattern of increased TLR signaling and decreased TCR signaling across all cardiovascular conditions. Our data also suggests that DNA methylation is a critical regulator of TCR and BCR signaling in these cardiovascular conditions. This data serves as a foundation for the study of novel gene networks and critical hub genes regulating innate and adaptive immunity in complex cardiovascular diseases.

The immune system plays a critical role in orchestrating and effecting the chronic inflammatory response associated with atherosclerotic diseases. Stimulation of the innate immune system by damage or pathogen-associated patterns produces an immediate but non-specific inflammatory response via TLR signaling. Our data suggests that TLRs are chronically activated in cardiovascular disease, associated with a decrease in TCR and BCR expression in the peripheral blood. This expression pattern may be the result of chronic tissue damage, attempts to resolve chronic inflammation, and decreased immune system reserve [16]. A hallmark of atherosclerotic disease is the accumulation of T cells within the atherosclerotic lesion where they play a direct role in plaque rupture [17]. With chronic inflammation occurring over decades, circulating T cells continuously migrate to the site of atherosclerotic lesions, resulting in a decrease of the T cell repertoire and expression in the peripheral blood, as shown by our data. To date, very little is known about how the adaptive immune system response changes in chronic cardiovascular disease. Given our data, we hypothesize that over time the adaptive immune system will respond differently to chronic inflammatory signals to conserve energy and maintain tissue homeostasis, resulting in immune senescence, as seen in several other chronic inflammatory diseases and cancer [18–20]. During the last two decades, various reports confirm that T cells are functionally impaired in several chronic inflammatory diseases, displaying a decreased expression of the TCR-ζ chain/CD3 complex [21–23]. Our data reveal a consistent pattern of down-regulation of the TCR-ζ chain/CD3 complex in all three diseases states, supporting the hypothesis that immune senescence is a pathophysiological consequence of chronic inflammation in atherosclerosis. It will be important to determine the effects of immune senescence on post-event immune suppression, as this will have implications for the treatment and recovery of IS or MI.

Global hypomethylation has been implicated in oncogenesis and autoimmune diseases [24,25]. DNA from CD4+ T cells is hypomethylated in aged mice contributing to higher Treg-mediated activity and increased immune suppression [20]. DNMT1 is responsible for the maintenance of DNA methylation, playing a critical role in epigenetic regulation of gene expression. DNMT1 deletion in the Treg lineage leads to lethal autoimmunity [26]. The role of T regulatory cells in IS and atherosclerosis is currently under significant investigation, primarily for their potential contribution to autoimmunity. In this study, DNMT1 expression was down-regulated in atherosclerosis, MI, and IS patients (signifying hypomethylation) and positively correlated with expression of markers in TCR signaling (i.e, CD81, CD3). Our findings suggest that the severity and duration of atherosclerosis contributes to changes in DNMT1 expression. We found a positive correlation between DNMT1 expression and TCR/BCR expression. As DNMT1, a global marker of methylation, may regulate adaptor proteins, cytokines and chemokines that play a role in activating T and B cell responses, given our results, it is also of interest to determine whether crosstalk between TLR and TCR signaling occurs through epigenetic modification of effector genes, such as CD3 or cytotoxic T-lymphocyte antigen 4 (CTLA4). Future studies are needed to determine the functional pathways mediating these relationships.

We recognize there are limitations to our current study, most notably the lack of functional data to validate our findings. However, WGCNA is a powerful bioinformatics tool that has been used to successfully identify functional mechanisms of various diseases. Our data supports current literature, suggesting significant relationships between epigenetic modification and immune senescence in chronic cardiovascular disease. We provide no evidence for a similar immune response in the brain. Thus, it will be important to determine how T-cell responses in the brain differ under various CVD states. IS and MI have systemic immune consequences and future studies should consider the contributions of the immune system and long-standing atherosclerosis on treatment. Our data provides many functional therapeutic targets to study at the bench.

## Conclusion

For the first time we compare blood transcriptional profiles of major immune signaling pathways among asymptomatic atherosclerotic disease, IS, and MI. We report significant overlap among TLR, TCR, and BCR signaling in these three pathologically similar but clinically different disease states and suggest that DNA methylation plays a central role in mediating the immune response to acute IS and MI. Our findings have the potential to guide functional studies of immune dysregulation to improve clinical risk stratification and development of novel treatment strategies for these conditions.

## Figures and Tables

**Table 1:** Significant enrichment of differentially expressed genes in each immune signaling pathway for the three diseases conditions. Number in denominator is total number of genes mapped to the pathway from the BioSystem database. Number in the numerator is the genes with P<0.05 when comparing disease vs control (atherosclerosis or IS) or 7 days vs 28 days after MI.

Pathway	Atherosclerosis	Ischemia Stroke	Myocardial Infarction
TLR	39/104 (38%)	48/102 (47%)	30/100 (30%)
TCR	53/104 (51%)	55/101 (54%)	55/103 (53%)
BCR	41/72 (57%)	42/70 (60%)	31/71 (43%)
